# Phenotypic Spectrum and Molecular Basis in a Chinese Cohort of Osteogenesis Imperfecta With Mutations in Type I Collagen

**DOI:** 10.3389/fgene.2022.816078

**Published:** 2022-01-28

**Authors:** Peikai Chen, Zhijia Tan, Hiu Tung Shek, Jia-nan Zhang, Yapeng Zhou, Shijie Yin, Zhongxin Dong, Jichun Xu, Anmei Qiu, Lina Dong, Bo Gao, Michael Kai Tsun To

**Affiliations:** ^1^ Department of Orthopaedics and Traumatology, The University of Hong Kong-Shenzhen Hospital (HKU-SZH), Shenzhen, China; ^2^ School of Biomedical Sciences, Li Ka Shing Faculty of Medicine, The University of Hong Kong, Hong Kong, China; ^3^ Department of Orthopaedics and Traumatology, Li Ka Shing Faculty of Medicine, The University of Hong Kong, Hong Kong, China

**Keywords:** osteogenesis imperfecta, targeted amplicon sequencing, COL1A1, COL1A2, bisphosphonate, bone mineral density

## Abstract

Osteogenesis imperfecta (OI) is a rare inherited connective tissue dysplasia characterized with skeletal fragility, recurrent fractures and bone deformity, predominantly caused by mutations in the genes *COL1A1* or *COL1A2* that encode the chains of type I collagen. In the present study, clinical manifestations and genetic variants were analysed from 187 Chinese OI patients, majority of whom are of southern Chinese origin. By targeted sequencing, 63 and 58 OI patients were found carrying mutations in *COL1A1* and *COL1A2* respectively, including 8 novel *COL1A1* and 7 novel *COL1A2* variants. We validated a novel splicing mutation in *COL1A1*. A diverse mutational and phenotypic spectrum was observed, coupling with the heterogeneity observed in the transcriptomic data derived from osteoblasts of six patients from our cohort. Missense mutations were significantly associated (χ^2^ *p* = 0.0096) with a cluster of patients with more severe clinical phenotypes. Additionally, the severity of OI was more correlated with the quality of bones, rather than the bone mineral density. Bone density is most responsive to bisphosphonate treatment during the juvenile stage (10–15 years old). In contrast, height is not responsive to bisphosphonate treatment. Our findings expand the mutational spectrum of type I collagen genes and the genotype-phenotype correlation in Chinese OI patients. The observation of effective bisphosphonate treatment in an age-specific manner may help to improve OI patient management.

## Introduction

Osteogenesis imperfecta (OI), also known as “brittle bone disease”, is a group of hereditary connective-tissue disorders with an incidence of ∼1:15,000 births (Lindahl et al., 2015). Patients with OI are more susceptible to long bone fractures and generally characterized by various degrees of bone deformity, blue sclerae, dentinogenesis imperfecta, scoliosis, hearing loss in young adulthood and decreased pulmonary function (Forlino et al., 2011; Marini et al., 2017). The spectrum of clinical manifestation ranges from mild to severe. The original grading system proposed by Sillence *et al.* classifies OI patients into four categories (Sillence et al., 1979), based on clinical and radiographic characteristics. Type I OI is the mildest form characterized by increased bone fragility and blue sclerae without obvious deformity. Type II OI causes perinatal lethality with intrauterine fracture. Patients with type III OI present multiple fractures and progressive skeletal deformities during the neonatal period. Type IV OI shows variable degrees of bone deformity with a severity intermediate between type I and III. This classical grading system has been re-defined, adding type V OI characterized by unique interosseous ossification, radial head dislocation and hyperplastic callus formation (Van Dijk and Sillence, 2014).

Since early 1980s, OI has been known as an autosomal dominant disease caused by mutations in *COL1A1* and *COL1A2*, which encodes the α1 and α2 chains of type I collagen, the most abundant extracellular matrix in bone, tendon and skin (Forlino et al., 2011). With the development of next-generation sequencing (NGS), new genes have been identified with different inheritance patterns. To date, over 19 OI causative genes have been identified, with functions covering bone mineralization, collagen modification, crosslinking and osteoblast differentiation (Forlino and Marini, 2016; Marini et al., 2017; Moosa et al., 2019; van Dijk et al., 2020).

Autosomal dominant variants in *COL1A1* and *COL1A2* are the most prevalent mutations causing OI (Zhytnik et al., 2019). Type I collagen consists of two α1 and one α2 chains, each of which contains a triple-helical domain composed of Gly-X-Y repeats flanked by N and C terminal pro-peptides. The heterotrimer is assembled from C-terminal toward N-terminal, secreted from the endoplasmic reticulum, and finally cleaved by proteinases (Marini et al., 2017). Mutations resulting in quantitative change of type I collagen cause a mild OI phenotype. Such haploinsufficiency is usually caused by nonsense, frameshift or splicing mutations. In contrast, qualitative mutations alter the structure of type I collagen and weaken the connective tissues, leading to more severe forms of OI. Substitution of glycine with a bulkier or charged residue within the Gly-X-Y tripeptide repeat is the most common mutation disrupting the triple-helical assembly (Van Dijk and Sillence, 2014; Forlino and Marini, 2016; Marini et al., 2017; Tournis and Dede, 2018).

Mutation spectrums on autosomal dominant OI have been established in large cohorts of Swedish (Lindahl et al., 2015), Canadian (Bardai et al., 2016), Indian (Mrosk et al., 2018), Italian (Maioli et al., 2019), Japanese (Higuchi et al., 2021) and Chinese populations (Li L. et al., 2019; Xi et al., 2021), highlighting the genetic heterogeneity of OI. However, the relationship between clinical manifestations and genetic mutations, and the mutational spectrum of type I collagen remain to be further explored. *COL1A1* and *COL1A2* contain 51 and 52 exons spanning genomic regions of 18 and 38 kb respectively. More than 1,065 (Last update: Nov 18, 2020) and 612 unique (Last update: Nov 19, 2020) mutations have been respectively identified in *COL1A1* and *COL1A2* loci (http://www.le.ac.uk/ge/collagen/). In the present study, clinical manifestations and genetic variants were analysed from 187 Chinese OI patients, with the intention to further expand the mutational spectrum of type I collagen, and better establish the correlation between genotype and phenotype in OI patients. Analyses of transcriptomic data from osteoblasts with different dominant mutations further reflected the heterogeneity of OI patients.

## Materials and Methods

### Subjects

This study was approved by the Institutional Review Board of the University of Hong Kong–Shenzhen Hospital. In all, 187 patients diagnosed as OI in Department of Orthopaedics and Traumatology, the University of Hong Kong-Shenzhen Hospital (HKU-SZH, a tertiary general hospital in China) were included in this study. Informed consent was obtained for all patients or legal guardians of children under 18. Detailed medical history and physical examination were assessed and collected by clinicians. Peripheral blood was obtained for genetic test. Skeletal samples were collected for analyses after osteotomy operation based on availability.

### BMD and Selection of Control Groups

To assess the growth curve of OI patients under the influence of bisphosphonate treatment, in terms of height, weight and bone mineral density (BMD), we retrieved a set of age and gender-matched controls for such information from the hospital information system. The BMD were measured by the Discovery DXA system (Hologic Inc., Massachusetts) at HKU-SZH. The total hip and total spine (the lumbar regions combined) BMDs were used. Weight and height of each BMD measurements were also collected. If multiple scans of the same measurements were available, the maximum shall be taken.

To begin with, the 187 OI patients were divided into 5-year age groups and further separated according to gender, starting from 0–5 years as the first group. We randomly selected BMD data from non-OI records as controls. However, there is a strong age disparity between the OI and non-OI BMD records, with OI records being much younger than non-OI ones. For example, 664 and 92 records of measurements were retrieved from individuals below 20 y/o for OI and non-OI, respectively. On the other hand, 67 and over 20,000 measurements were obtained for those aged above 20, in the two same groups, respectively. To cope with this, we developed an age- and gender-stratified selection procedure. First, the ratio of non-OI to OI records in the 15–20 age-group was about 1:4 for both genders and dropped significantly afterwards. Next, we used this ratio to determine the optimal number of non-OI control to be 12 for the hip and 26 for the spine in each group. As such, we used all the non-OI data below 20 as controls, and randomly select another 12 and 26 records per each 5-year age-group from the non-OI hip and spine respectively, for those aged above 20.

The non-OI data were fitted with LOESS (Local Polynomial Regression Fitting) regression with default parameters using R function loess (), to produce a normal growth curve, denoted as f() . For any successive two readings of the same individual, a normal growth slope was calculated: $k=\left[f\left(t_{2}\right)-f\left(t_{1}\right)\right]/\left(t_{2}-t_{1}\right)$ . The actual growth slope was calculated $k'=\left(b_{2}-b_{1}\right)/\left(t_{2}-t_{1}\right)$ , where $b_{2}$ and $b_{1}$ are either BMD or height readings at $t_{2}$ and $t_{1}$ , respectively, with $t_{2}>t_{1}$ . The angle α was calculated as $\alpha =\tan^{-1}\left(k^{'}/C^{'}\right)-\tan^{-1}\left(k/C\right)$ , where $C^{'}$ and C are normalising scaling factors to ensure that angles for BMD and height are comparable.

### Targeted Amplicon Sequencing

**FIGURE 1:**
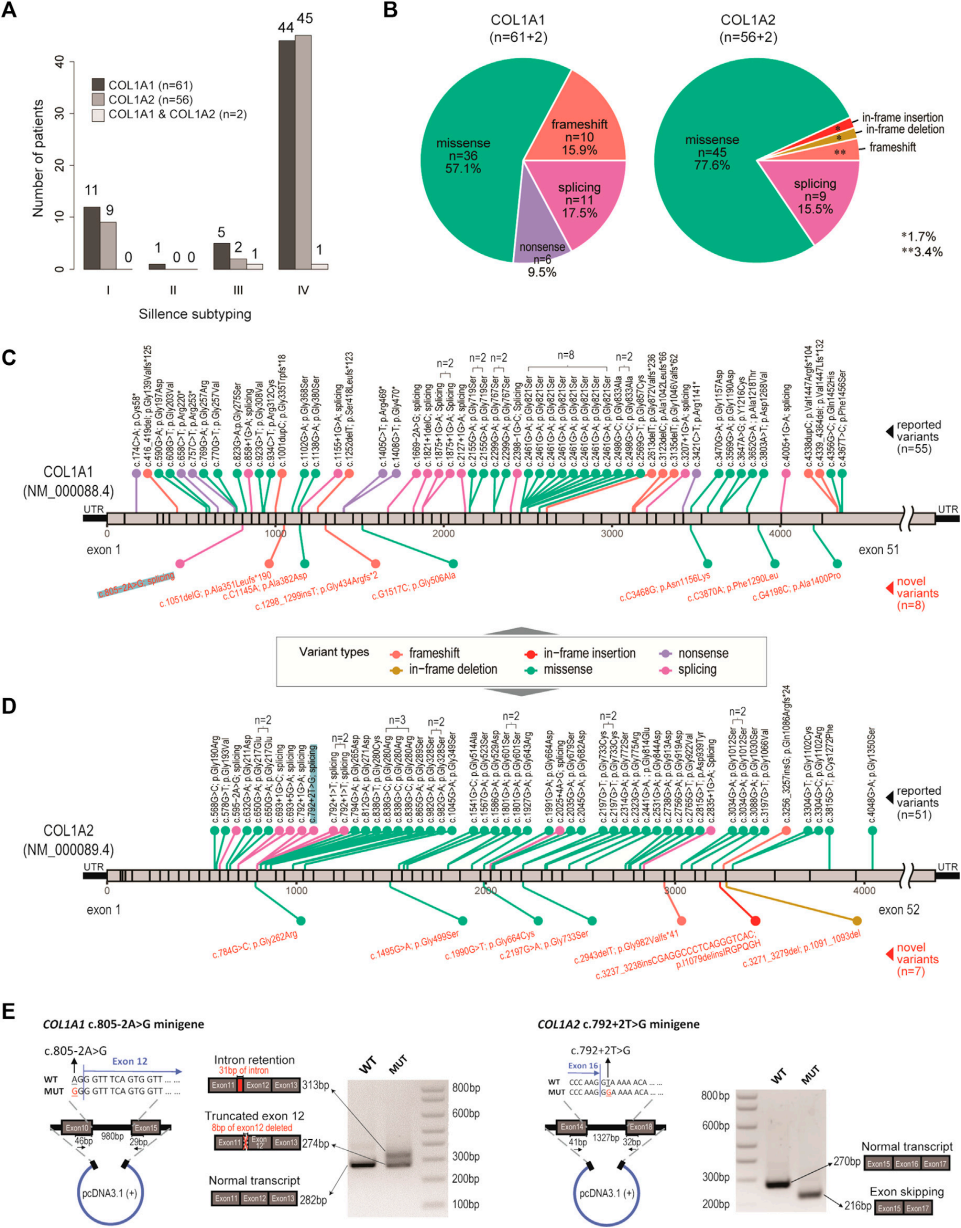
Genetic spectrum in our OI cohort. **(A)** Bar charts showing the distribution of OI cases, with respect to genotypes and clinical subtyping (Sillence scales). **(B)** Pie charts showing the distribution of variant types, with respect to genotypes. **(C)** Diagram showing location and mutation information of the pathogenic variants identified in COL1A1. n number indicates number of recurrences in the cohort. Black texts indicate reported variants. Red texts indicate novel variants. **(D)** Diagram showing location and mutation information of the pathogenic variants identified in COL1A2. n number indicates number of recurrences in the cohort. Black texts indicate reported variants. Red texts indicate novel variants. **(E)** Validation of a novel splicing variant (COL1A1, c.805–2A > G) and a positive control (COL1A2, c.792+2T > G), which were also highlighted in **(C)** and **(D)**.

DNA samples isolated from peripheral blood were subjected to targeted amplicon sequencing of *COL1A1* and *COL1A2* (Bybee et al., 2011). Libraries were prepared by a two-stage PCR process and incorporated with a unique 8-bp index for sample-specific barcoding, allowing all samples to be mixed for library purification and sequencing in a single run. Sequencing was performed on the NovaSeq 6000 system (Illumina) with a 150bp paired-end protocol in DynastyGene Co. (Shanghai) and aligned to GRCh37/hg19 genome reference. The GATK toolkit (version 4.0.4.0) (McKenna et al., 2010) was then used to call the variants from the aligned BAM files. The results were annotated by SNPeff (Cingolani et al., 2012) and ANNOVAR (Wang et al., 2010), and deposited in VCF (variant calling format) files to be reviewed by our team of clinicians and geneticists. Qualitative mutations were defined as missense variants, and quantitative as non-missense ones. The variants were visualized (Figures 1C,D) using custom scripts, with exon coordinates for *COL1A1* (NM_000088.4) and *COL1A2* (NM_000089.4) obtained from NCBI.

### Minigene Splicing Assay

The minigene splicing assay was performed to assess the effects of two intronic mutations in *COL1A1* (c.805–2A > G) and *COL1A2* (c.792+2T > G) on mRNA splicing. Target genome DNA fragments containing the mutated intron and the flanking upstream and downstream genome regions (exon10 to exon15 in *COL1A1* and exon14 to exon18 in *COL1A2*) were cloned into pcDNA3.1 (+) vector at *EcoRI* and *NotI* sites. Point mutations were generated by site-directed mutagenesis and validated by Sanger sequencing. Purified *COL1A1* and *COL1A2* minigene constructs were transfected into HEK293T cells using polyethylenimine. RNA was extracted from transfected cells by TRIzol reagent (Invitrogen) after 24-h, and reversely transcribed into cDNA using PrimeScript RT reagent kit with gDNA Eraser (TakaRa). Spliced mature mRNA fragments can be amplified from the cDNA with specific *COL1A1* (forward: 5′-TGGA AAA​CCT​GGT​CGT​CCT​GGT​GA-3′, reverse:5′-CCAGTAGCACCATCATTTCCACGA-3′) and *COL1A2* (forward: 5′-TTC​CTG​GTG​AGA​GAG​GAC​GTG​TTG-3′, reverse:5′-CACCAGT AAG​GCC​GTT​TGC​TCC​A-3′) primers. Amplified PCR products were separated on 2% agarose gel and purified by MiniBEST Agarose Gel DNA Extraction Kit V4.0 (TaKaRa). The specific splicing pattern was determined by subsequent Sanger sequencing.

### Bone Histology

The skeletal samples collected after necessary operations were fixed in 4% paraformaldehyde and decalcified with 0.5M EDTA before embedding in paraffin. 6 µm sections were cut and mounted on glass slides. The rehydrated sections were stained with Goldner’s trichrome and visualised with Leica DM3000 microscope.

### Bulk RNA Sequencing

Skeletal specimens from 1) six patients with type I collagen mutations after operations (e.g. osteotomy), 2) a normal boy with humerus fracture and 3) a patient with leg length discrepancy were collected for osteoblast isolation. Soft tissues were removed completely and blood cells were washed away with PBS. Bone samples were then minced into small pieces and immersed in osteoblast culture medium (aMEM with 10% fetal bovine serum, penicillin [100 U/ml] and streptomycin [100 μg/ml]) for 1–2 weeks for osteoblast migration and proliferation.

Confluent cells were lysed with Trizol (Invitrogen) and total RNA were extracted according to manufacturer’s instruction. RNA concentration was measured by Qubit and RNA integrity was assessed using the Agilent 2100. A total amount of 2 µg RNA per sample was used as input material. Sequencing libraries were generated using VAHTS mRNA-seq v2 Library Prep Kit from Illumina following manufacturer’s recommendations and sequenced on an Illumina NovaSeq platform to generate 150 bp paired-end reads by a commercial company (Berry Genomics, Beijing).

### Bioinformatic Analyses of RNA Sequencing Data

Raw data (raw reads) of fastq format were firstly processed through primary quality control. Clean data were aligned to the reference human genome (GRCh38/hg38) and gene expression (FPKM values) was calculated for each transcript using the HISAT2 (Kim et al., 2015) and Cufflinks package (Trapnell et al., 2012). HTSeq package sequencing read count was calculated as described (Anders et al., 2015). Differential expression analysis between two conditions was performed using the cuffdiff tool in the Cufflink package. Differentially expressed genes were defined with adjusted *p*-value < 0.01 and the log2(Fold change) > 2. Genes with average values < 1 in both groups under comparisons were excluded.

### Statistics

Data presented are the averages with standard deviation. Statistical significance level was evaluated by student’s *t*-test (two-tailed, unpaired) between two groups. The difference with *p* < 0.05 was considered significant. Wilcoxon signed rank tests were used for testing if the normalised angles are positive (>0) in the BMD and height tracking data.

## Results

### Clinical Characteristics of Our OI Cohort

In all, 187 patients diagnosed with OI in the past 5 years were included. Familial information was collected and the patients were found to group into 175 unrelated families, where 167 families have one patient only and 8 (20 patients) have multiple (Supplementary Table S1). The patients were admitted to the Hospital for orthopaedic surgeries, drug treatment or physiotherapy, which include 113 males and 74 females with hospital admission ages ranging from 1 to 38 (median 11, IQR 7–17). Geographically, 88.5% of the cohort came from southern China (south of Yangtze River). According to Sillence classification, the cohort displayed predominantly moderate to severe features, with 24 (14.6%), 1 (0.6%), 20 (11.7%) and 125 (73.1%) patients classified as subtypes I, II, III and IV, respectively. Another 17 patients showed typical type V OI features, including radial head dislocation, interosseous ossification and hyperplastic callus (Hanagata, 2016). Fractures were frequently reported, with 140 (74.5%) patients reporting at least one prior fracture event on their admission/visit to the Hospital, with an average of 13.5 previous fractures per patient. About two thirds (122 out of 187) reported previous surgical treatments and 117 (62.5%) patients reported previous drug treatments, with pamidronate and zoledronate accounting for 26.5 and 73.5%, respectively. Only one patient was treated with denosumab. Physical inspections for typical OI traits, including blue sclerae, limb deformity, dentinogenesis imperfecta, scoliosis, joint laxity, flat feet, basilar invagination, etc. were also recorded, which shall be discussed in later sections.

### Targeted Sequencing Revealed Pathogenic Variants in *COL1A1* and *COL1A2*

By targeted amplicon sequencing covering the coding regions and exon-intron boundaries of *COL1A1* and *COL1A2* loci, 119 patients (63.6%) were found to be carrying pathogenic mutations on *COL1A1* (n = 61), *COL1A2* (n = 56) or both (n = 2) (Figure 1A; Supplementary Table S1). The other 68 patients (referred to as the OI-nonCOL1 group) shall be investigated in future, by an expanded gene-panel, copy number variation (CNV) analysis by MLPA or by whole-genome sequencing. Clinical subtypes of these 119 OI-COL1 patients were predominantly Types I and IV, with no detectable difference between the two affected genes (χ^2^ *p* = 0.84) (Figure 1A). Interestingly, the compositions of variant types are significantly different (χ^2^ *p* = 0.013) between individuals carrying *COL1A1* and *COL1A2*, with missense mutations representing 57.1 and 77.6% of all events in the two genes, respectively (Figure 1B). Frameshift and nonsense mutations were much more frequent in *COL1A1* (15.9 and 9.5%, respectively) than in *COL1A2* (3.4 and 0%, respectively); whereas variants affecting splicing were comparable (17.5% in *COL1A1* and 15.5% in *COL1A2*). For the two patients carrying mutations on both *COL1A1* and *COL1A2*, one (glycine substitution mutations in both genes) displayed type III features, and the other (alanine substitution on *COL1A1* and frameshift on *COL1A2*) was classified as type IV (Supplementary Table S2).

The positions and nature of these variants were displayed in the *COL1A1* and *COL1A2* locus chart, with 8 and 7 novel pathogenic mutations detected, respectively (Figures 1C,D). We noted that a “hotspot” missense mutation was present in 8 patients (*COL1A1*, c. 2461G > A), though four of them came from a consanguineous family (Supplementary Table S1). A number of other recurrent variants can be found in both genes (Figures 1C,D). A novel splicing mutation (*COL1A1*, c.805–2A > G) was selected to validate its deleterious effect on mRNA splicing.

The *COL1A2* (c.792+2T > G) splicing mutation was selected as a positive control (Zolezzi et al., 1995). Splicing mutations causing premature termination may produce mild phenotypes, while variants resulting in reading frame shift and impaired triple helix structures may be associated with more severe phenotypes (Marini et al., 2007). The patient with c.805-2A > G (*COL1A1*) variant displayed severe skeletal deformity (type IV) including scoliosis and low bone density in the spine (Z score: −4.8). The patient with c.792+2T > G (*COL1A2*) variant showed mild type I features (Supplementary Table S2). The minigene assay showed that the mutation (*COL1A1*, c.805-2A > G) resulted in mRNA splicing abnormalities, including intron retention and exon truncation (Figure 1E). Consistently, exon 16 skipping was observed in the *COL1A2* (c.792+2T > G) variant (Zolezzi et al., 1995). These data expanded the genetic spectrum for OI patients affected by *COL1A1/2* mutations.

### Clinical, Histological and Molecular Diversification Coupled With Genetic Spectrum

**FIGURE 2:**
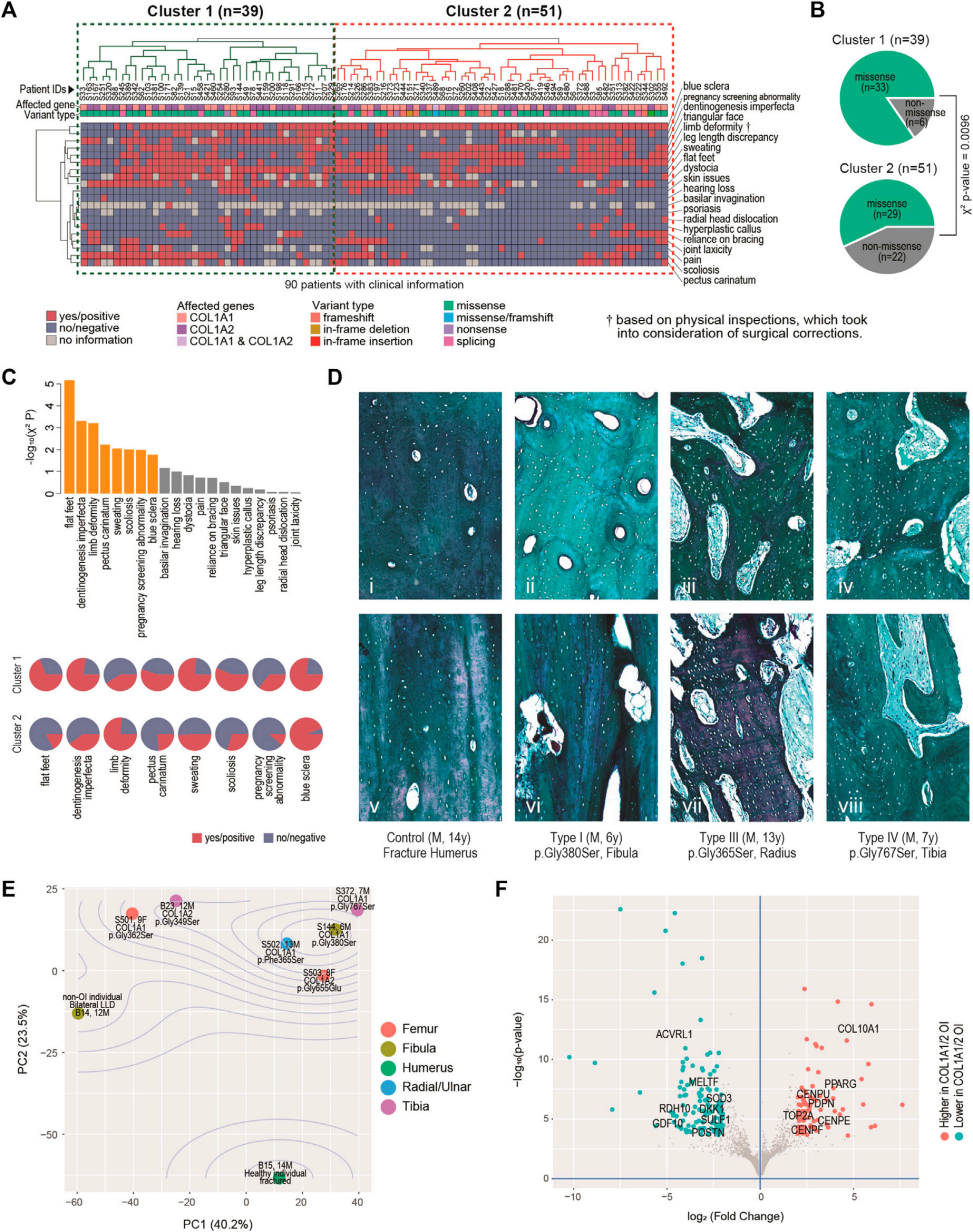
Clinical, histological and molecular phenotypes. **(A)** Heatmap showing clinical characteristics for 90 patients affected by COL1A1 or COL1A2, and with available information. Euclidean distance and complete linkage were used in constructing the dendrograms. **(B)** Enriched types of variants in the identified clusters. **(C)** Top: bar charts showing the clinical traits most correlated with the two clusters. Bars in orange have *p* < 0.05. Bottom: arrays of pie charts showing the percentages of positive traits in each of the two clusters, for the 8 clinical traits that have *p* < 0.05. **(D)** Analyses of bone histology. Goldner trichrome staining of skeletal samples from control individual (n = 1) (i, v), proband with type I OI (n = 2) (ii, vi), proband with type III OI (n = 2) (iii, vii) and proband with type IV OI (n = 3) (iv, viii) harbouring different mutations in COL1A1. Haversian structure was shown by transaxial sections (i-iv). Collagen matrix alignment was shown by sagittal sections (v-viii). **(E)** Diagram of Principal component (PC) analyses showing the sample-sample relationships in a set of eight bulk transcriptome data derived from osteoblasts of six OI and two non-OI patients. Percentages on axes indicate fraction of variance explained. **(F)** Volcano plot showing the differentially expressed genes between the six OI samples with mutations on COL1A1/2 and the two controls.

Twenty critical clinical traits for OI patients, including blue sclera, dentinogenesis imperfecta, hearing loss, joint and skeletal issues, etc. were collected from 90 of the 119 patients. Based on the hierarchical clustering of these data, two clusters of patients can be identified, one with 39 patients (Cluster 1) and the other with 51 (Cluster 2) (Figure 2A). Visually, more negative entries (in blue) were observed in Cluster 2. Interestingly, although no association was found between the genotypes and the two clusters (χ^2^ *p* = 0.11); there is a strong tendency (χ^2^ *p* = 0.0096) of higher missense mutations in Cluster 1 (33/39, 84.6%) than in Cluster 2 (29/51, 56.8%) (Figure 2B).

The 20 traits contributed differently to the two-cluster patterns, with 8 of them being significantly correlated with the two-cluster classification (χ^2^ *p* < 0.05) (Figure 2C, top chart). Of note, ratios of flat-fleet, dentinogenesis imperfecta, pectus carinatum, over sweating, scoliosis and pregnancy screening abnormality were higher in Cluster 1 than in Cluster 2, indicating overall more severe clinical phenotypes in the Cluster 1 (Figure 2C, bottom chart). Limb deformity was found to be lower in Cluster 1. Limb deformity was not based on pre-orthopaedic correction conditions, but on physical inspection upon the patients’ presentation to the physicians. Lower limb deformity rate in Cluster 1 may in fact suggest a higher chance of prior orthopaedic corrections and thus a more severe prior deformity condition in this cluster. The lower rate of blue sclerae in cluster 1 was also found, consistent with the fact that blue sclera was typically more prevalent in less severe OI (Marini et al., 2017).

The mechanical strength of bone is determined not only by the degree of mineralization but also the alignment of collagen fibres. To understand the molecular basis correlated with the severity of OI patients, we further characterized the bone geometrical property in the control and affected individuals. Transaxial sections indicated that the size and number of haversian canals and resorption cavities were significantly increased in the cortical bones from type I OI patients (Figure 2Dii), when compared to control samples (fracture humerus, 13 years/o, male) with compact haversian structure (Figure 2Di). The structure became even worse in type III and type IV patients, showing more severe clinical features (Figures 2Diii,iv). Bone histology from sagittal sections also indicated similar trends. The control sample showed condensed and organized lamellar pattern (Figure 2Dv), whereas the bones from affected individuals displayed increasing porosity with disorganized collagen alignment from type I to type III and IV (Figures 2Dvi–viii), suggesting that the severity of clinical manifestations was positively correlated with the degree of abnormal bone geometry.

To build up more connections between genetics and clinical/histological changes, we conducted bulk transcriptomic profiling on osteoblasts derived from six OI individuals and two non-OI samples. Principal component analyses (PCA) showed that the first two PCs captured 63.7% of the data variance in combination (Figure 2E). The two non-OI samples lie on one side (lower left) of the PCA chart, while all the six OI samples lie on the other side, with the fractured sample from normal individual lying furthest from the OIs. The OI group also showed some degree of variation, although the majority of osteoblasts were isolated from patients with glycine substitution mutation (Figure 2E). The transcriptomic variation may partially explain the diversity of clinical features among OI patients. To identify the gene expression changes commonly affected by type I collagen mutations, we went on to detect the differentially expressed genes (DEGs) between the two groups. At FDR<0.01 (false discovery rate) and log_2_ (fold change) > 2, we detected 85 DEGs higher and 121 DEGs lower in the OI-COL1 group, as compared with the two non-OIs (Figure 2F; Supplementary Table S3; Supplementary Figure S1). It was noteworthy that *COL10A1* and *PPARG* were listed among the genes higher in the OI-COL1 group. COL10A1 is a marker for hypertrophic chondrocytes, which can become osteoblasts (Yang et al., 2014; Tsang et al., 2015). *PPARG* encodes a transcription factor PPARγ critical for adipogenesis (Rosen, 2005). It was reported that osteogenesis and adipogenesis were counteracting forces in normal bone formation, and compromised osteogenesis may lead to more active adipogenesis (Akune et al., 2004; Zhang et al., 2006). The increased expression of *COL10A1* and *PPARG* observed in OI samples may suggest the change of cell fate in the mutant osteoblasts. The genes lower in OI-COL1 group included osteochondrogenic marker *GDF10* (Kratochvilova et al., 2021), indicating reduced bone formation activities in the OI group.

### Compromised Weight, Height and BMD in OI Patients

**FIGURE 3:**
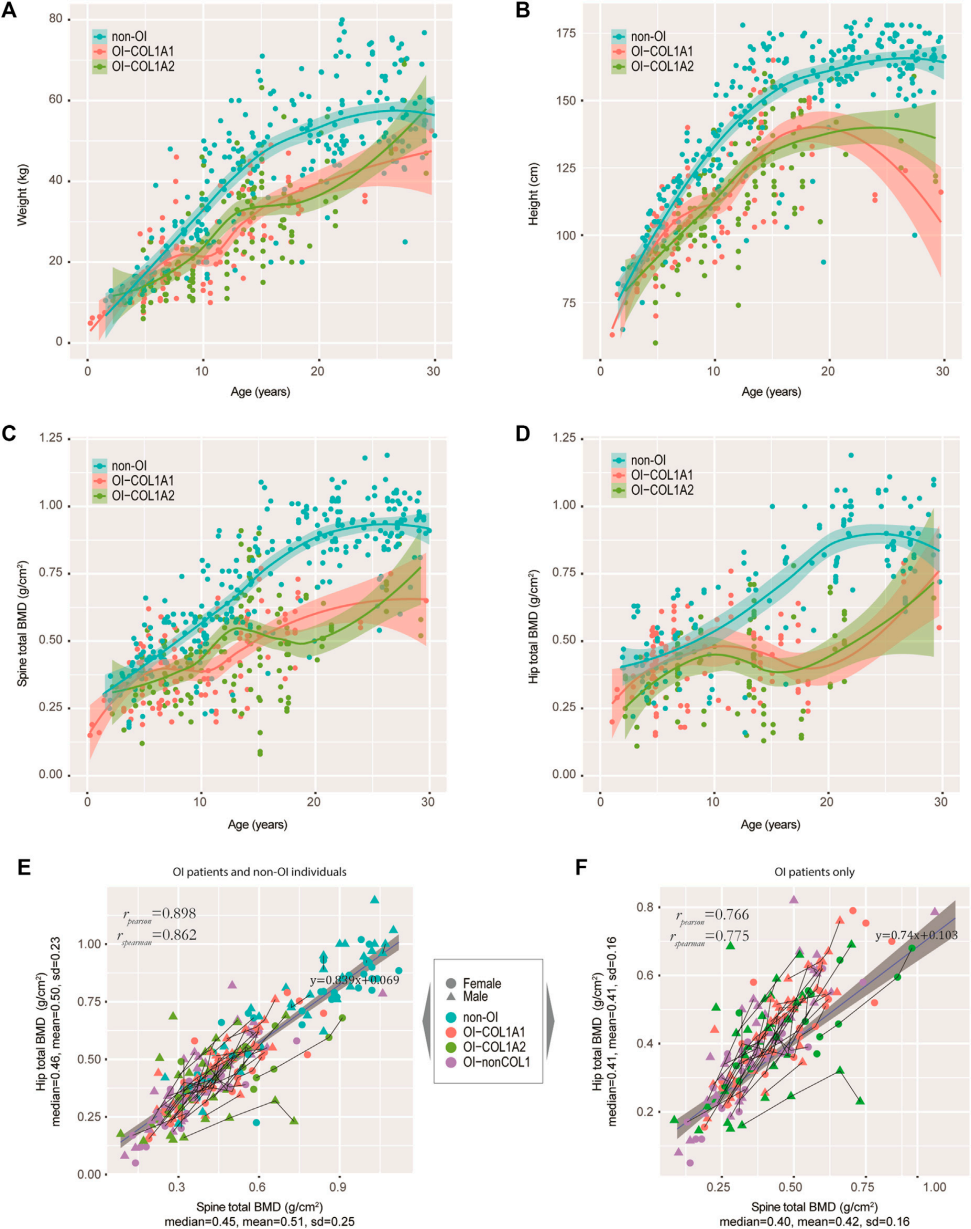
Tracking the weight, height and BMD in our OI cohort. **(A−D)** Growth curves of weight **(A)**, height **(B)**, spine BMD **(C)**, and hip BMD **(D)** were tracked. Non-OIs were randomly retrieved from our hospital information system with gender and aged matched (per age group), and served as controls for comparison. Each dot represents one measurement for one individual. **(E)** Scatter plot showing the correlation between spine and hip BMDs in our OI and non-OI cohorts combined. **(F)** Scatter plot showing the correlation between spine and hip BMDs in our OI cohort only. Line segments indicated measurements of the same individuals.

It is of interest to track several key growth indicators, including weight, height and BMD in OI-COL1 patients with consideration of age and gender, in comparison with non-OI individuals. Since many OI patients have metal rodding inside their limbs, BMD readings in these sites may not be suitable for analyses. Instead, the spine BMD was often used, although readings on the hip may also be valuable. We first carefully selected a set of patients without OI features as controls (referred to as the non-OIs) (Methods). Majority of the OI patients had BMD measurements (181 out of 187), with matching height and weight measurements. Figures 3A,B showed the weight and height readings for the four groups of individuals. Statistically, the readings of weight and height from OI patients were significantly lower than the non-OI for the age-groups 15–20, 20–25 and 25–30 years of the females; and for age-groups 10–15, 15–20 and 25–30 years of the males (Supplementary Figure S2A,B). Not much difference was found among the three OI groups in both genders. For height, although in both genders, the divergence starts as early as the 5–10 years group, the biggest gap occurs from the 15–20 group, which was maintained into the 25–30 years group (Figure 3B; Supplementary Figure S2C,D). The total BMD in the spine and in the hip showed a similar trend, where the gap between non-OI and OI widens from the 10–15 age-group and onwards (Figures 3C,D; Supplementary Figure S2E,F). Indeed, the spine and hip BMD showed a strong correlation (Pearson correlation *r* = 0.898) (Figure 3E), although this correlation dropped slightly (Pearson correlation *r* = 0.766) in the OIs (Figure 3F). Interestingly, the growth behaviours between *COL1A1* and *COL1A2* patients showed much less divergence, in weight, height and BMD.

We also asked if mutation types (qualitative or quantitative, defined in Methods) may impact the growth curves of height. We fitted the data with a non-linear mixed-effect model using SITAR (Cole et al., 2010), using height data from individuals with more than 4 separate measurements and spline degree of freedom of 3, just to avoid model unidentifiability. We found that the patient-specific random effects for qualitative and quantitative mutations were on average 4.1 cm below and 3.1 cm above population average, respectively. This is consistent with our earlier findings that missense mutations have more negative phenotypes (Figures 2A–C).

### Tracking the Impact of Bisphosphonates on BMD Revealed Age-specific Improvement

**FIGURE 4:**
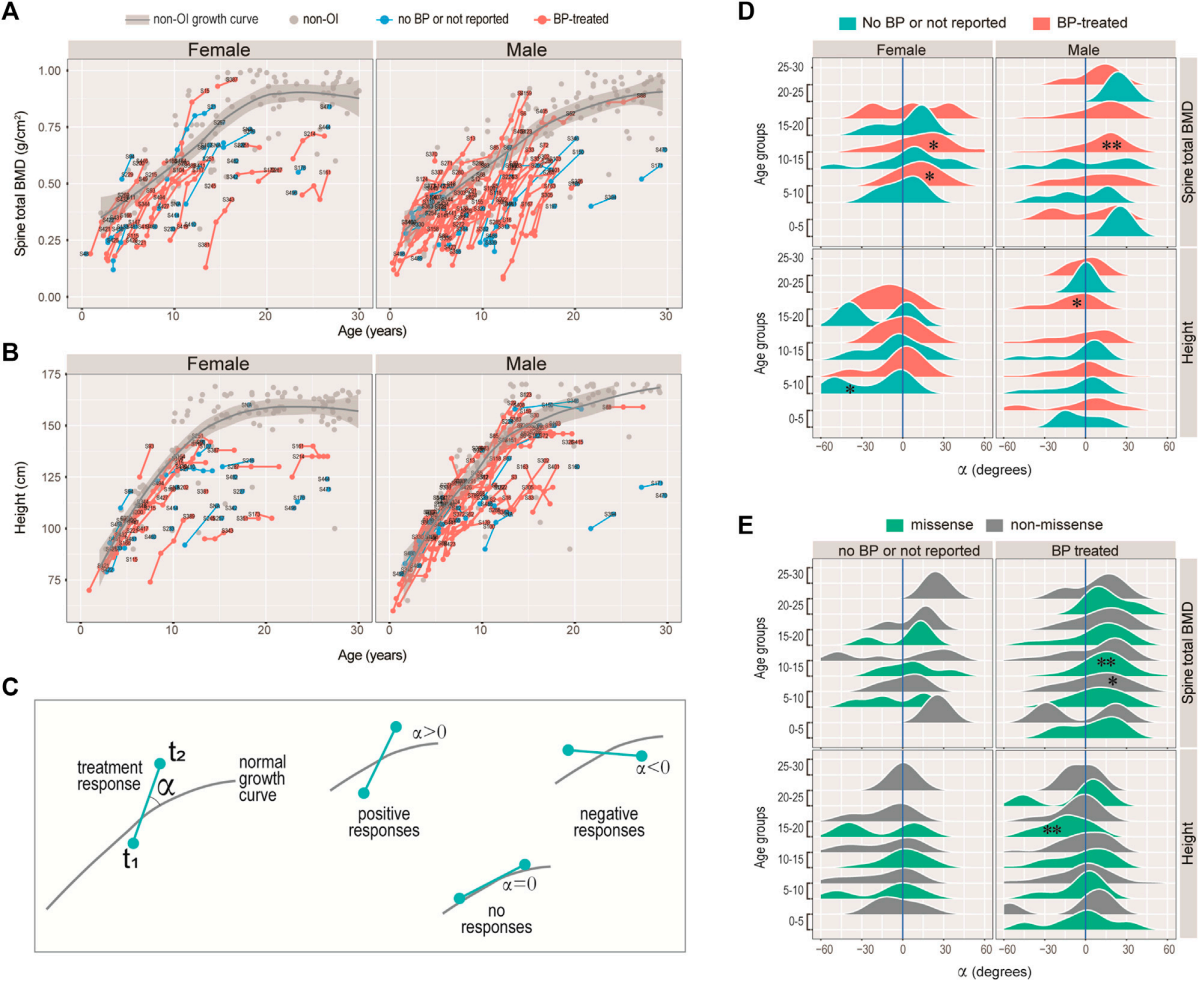
Responses of bisphosphonate (BP) treatment. **(A–B)** Growth curves for spinal BMD **(A)**, and height **(B)** for the female and male patients with *COL1A1/2* variants in our cohort. Line segments indicated measurements of the same individuals. Text labels were anonymized patient IDs. **(C)** Diagram illustrating the strategy measuring BP responses. The angle *α* was measured between the slope of the consecutive measurements and the tangential slope in the controls. A positive angle indicates stronger growth rate as compared with the controls, and a negative one indicates otherwise. **(D–E)** Ridge plots showing the BP responses of spinal BMD and height with respect to sex, BP-history and age-groups **(D)**, or to variant type, BP-history and age-groups **(E)**. *: Wilcoxon test *p* < 0.05; **: *p* < 0.01; unmarked box: no significant difference.

Among the 108 (out of 119) OI-COL1 and 60 (out of 68) OI-nonCOL1 patients with BMD data, 281 and 178 records were retrieved, amounting to an average of 2.6 and 3.0 records per individual, respectively. About 3/4 of these patients reported being treated by bisphosphonates (BP), with 72.1, 75.6 and 78.6% for the COL1A1, COL1A2, and OI-nonCOL1 groups, respectively (Supplementary Figure S3A). On average, an OI patient reported receiving 4.6 prior BP treatments (IQR 2.0–6.0) (Supplementary Figure S3B). Based on current records, 64 patients had two or more BMD records for the spine, 54 of which treated with BP. Given the linear relation between spine and hip (Figures 3E,F) and the quadratic relation between height and weight (Supplementary Figure S3C), we focused on the spine BMD and height for treatment response analyses. Line segments linking successive spine BMD (Figure 4A) and height (Figure 4B) readings of the same individuals were shown, with grey dots indicating the non-OI readings of BMD and height, and their fitted curves (Methods) with standard error band.

Intuitively, we noticed that although the BMD and height readings tend to fall below the non-OI fitted growth curves, the BMDs’ slopes appeared to be steeper than the non-OI fitted curve, whereas the heights’ slopes were more or less tangent to the curve. We asked if BMD was more responsive to BP than height, and if the response was age-dependent. Nonlinear mixed effect models were frequently used to fit the growth curves (Cole et al., 2010; Susman et al., 1998), where both the deviation from normal and transient velocity (growth rate) can be estimated. Unfortunately, extensive data points per individual are needed to avoid model unidentifiability. Alternatively, we used an *ad hoc* approach to measure the normalised angle (Methods) between the slope of the successive readings and that on the fitted curve (Figure 4C). A positive angle would indicate a growth rate faster than normal (i.e. non-OI), a negative angle would mean a slower response and a zero angle for no response (Figure 4C). Figures 4D,E showed the angle, with respect to data type (BMD or height), gender, and treatment history. It appeared that the angles for height were either not different from zero, or significantly lower (lower panels of Figures 4D,E). In particular, height growth appeared to be significantly lower in the BP-treated OI in the 15–20 age-group, which was consistent with a mouse study showing BP-treatment inhibits long bone growth (Evans et al., 2003). In comparison, the angles for spine BMD showed a strong tendency to deviate to the right of the zero axis (upper panels of Figures 4D,E). In addition, the significantly positive angles tended to occur in the 5–15 age-groups. These results suggest that BP treatment may not be helpful to improving height, but it does improve bone density, particularly when administered in the juvenile age-groups (5–15 years).

## Discussion

Considering the clinical complexity and genetic heterogeneity of OI, NGS-based genotyping enables precise diagnosis and genetic counselling for OI cases. In the current study, we analyzed the pathogenic variants in the type I collagen of 187 patients diagnosed with OI, and revealed the correlation between genotype and phenotype. Our results expand the clinical features, genetic spectrum and molecular basis to the Chinese cohorts of OI (Zhang et al., 2016; Liu et al., 2017; Li L. et al., 2019; Li L.-J. et al., 2019; Xi et al., 2021), with a particular focus on the OI population from southern China. OI is generally considered a monogenic skeletal disorder. At current stage, we identified a total of 102 unique mutations in *COL1A1* and *COL1A2*, including 15 novel mutations. Variants in type I collagen accounted for the majority of all patients (63.6%, 119 out of 187). With regard to the proportion of collagen-related OI in different cohorts, the detection rates were high up to 85–90% in Italian (Maioli et al., 2019), Canadian (Bardai et al., 2016) and Japanese (Higuchi et al., 2021) populations. It is noteworthy that type I mild OI predominates in these cohorts. While in the cohort from Indian with majority of moderate to severe OI, only 46% of OI cases were caused by mutations in *COL1A1* and *COL1A2*, and the moderate to severe form of OI accounts for a higher proportion (Mrosk et al., 2018). The deviation may lie in the bias of individuals with severe phenotypes to seek orthopaedic treatment, geographical isolation with higher risk of consanguineous history, genetic heterogeneity vulnerable to autosomal recessive defects, epigenetic, environmental, and other unidentified factors associated with the corresponding variability. Our cohort only consists of 16.8% type I mild OI (20/119), while moderate to severe OI types dominate the cohort. Compared with patients with autosomal dominant OI, those with recessive inheritance tend to show more severe skeletal deformities (Marini et al., 2017; Li et al., 2020). Population-based studies would be more representative to determine the OI mutations spectrum by removing the inheriting biases and hospital-based referral trends.

In accordance with the literature, mild OI cases in our cohort were caused both by quantitative and qualitative defects in type I collagen defects (Forlino and Marini, 2016). Although mild form of OI was generally caused by mutations in *COL1A1* and *COL1A2* (Bardai et al., 2016), we observed four patients without suspicious variants detected in type I collagen, where no record of extraskeletal features was known to us. No correlation was observed between the variant position and clinical manifestations, since mutations associated with mild to severe OI types were dispersed throughout the genomic loci of *COL1A1* and *COL1A2*. Interestingly, a frequent variant detected in Chinese patients (c. 2299G > A; p.Gly767Ser in *COL1A1*) (Xi et al., 2021) was also identified in our cohort. Another hotspot (c. 2461G > A; p.Gly821Ser in *COL1A1*) was present in 8 patients (belonging to five unrelated families). Interestingly, remarkable phenotypic variability ranging from moderate to severe was observed among individuals bearing this variant (Supplementary Figure S4). It was recently suggested that haploinsufficiency in familial cases may cause similar features, whereas structural abnormality results in higher phenotypic variability (Zhytnik et al., 2020).

Obtaining a precise diagnosis of OI in different populations expands the understanding of molecular basis in OI and improves the personalized care. Notably, no nonsense variants were identified in *COL1A2* loci, consistent with a previous study in Chinese OI cohort (Li L. et al., 2019). This bias may originate from the composition proportion of the heterotrimer (two α1 and one α2 chains), suggesting a significant difference between *COL1A1* and *COL1A2* where a decreased amount of the α2 chain causes minor interruption of type I collagen (Rauch et al., 2010). The proportion of missense mutations in *COL1A1* in our study was lower than reports in India (85.7%), Vietnam (67.6%), and Sweden (60.9%) (Stephen et al., 2014; Lindahl et al., 2015; Ho Duy et al., 2016), but was still the dominant variant in the spectrum. Two patients were found harboring compound mutations of *COL1A1* and *COL1A2*. One of them with two known pathogenic variants (c.590G > A in *COL1A1* and c. 650G > A in *COL1A2*) displayed severe phenotypes (type III), while the other patient with two novel variants (c.4918G > C in *COL1A1* and c.2943delT in *COL1A2*) showed moderate features (type IV). The variant c.2943delT in *COL1A2* was considered as the likely pathogenic factor because it was inherited from his father with OI, while the mutation c. 4918 G > C in *COL1A1* was considered as variant of uncertain significance (VUS) that requires further validation. In our study, we only identified a homozygous mutation in *COL1A1* (c. 3803A > T; p. Asp1268Val) leading to embryonic lethality, which was not found in the variants involved in the lethal outcome (Maioli et al., 2019). On the other hand, a lethal mutation in the same Italian cohort was found recurrent in our spectrum, which may further reveal the genetic heterogeneity among different ethnic populations.

The relationship between the genetic mutations and clinical severity is complex in OI patients. Glycine substitutions and splicing mutations are the dominant variants, leading to structural defects (qualitative) or quantitative change in type I collagen (Forlino and Marini, 2016). Glycine is the only amino acid small enough to fit into the restricted space of the helical centre. Glycine substitutions usually disrupt the helix stability and produce moderate-to-severe phenotypes (Marini et al., 2017), which was corroborated by the unbiased clustering analyses in our study. Splicing variants that cause reading frame shifted and impaired triple helix structures may result in severe phenotypes. Conversely, mutations resulting in premature termination may produce mild phenotypes (Marini et al., 2007), which may explain the different severities in the patients carrying novel splicing variants in our study. The patient with c.805–2A > G in *COL1A1* was classified in type III OI, while the patient with c.792+2T > G in *COL1A2* displayed type IV features.

Consistent with previous studies, blue sclera was associated with the mild form of OI, while dentinogenesis imperfecta was detected more in patients with moderate-to-severe features, suggesting the similarity of dentin and bone with regard to the composition in the extracellular matrix (Figure 2C) (Bardai et al., 2016; Li L. et al., 2019; Higuchi et al., 2021). Hearing loss, a common secondary feature of OI with mixed conductive and sensorineural deficiency, often develops between the second and fourth decades of adult patients (Hald et al., 2018). In the current study, only two probands encountered hearing loss, with much lower proportion than those reported in Chinese (7.5%) and European (24%) populations (Hald et al., 2018; Xi et al., 2021), which may be explained by the majority of paediatric patients in our cohort. Furthermore, four individuals presented with hearing impairment in adolescence and young adulthood, but the number of cases was insufficient for the assessment of genotypic associations (Higuchi et al., 2021). Consistently, the height of OI patients, particularly in the severe forms, was significantly lower than that of the normal population (Bardai et al., 2016; Li L.-J. et al., 2019), while there was no difference between patients with mutations in *COL1A1* and *COL1A2*.

Bisphosphonates, as the mainstay of pharmacological intervention for osteoporosis, are widely used to improve the BMD for OI patients, aiming to increase their bone mass and reduce the limb deformity (Sato et al., 2016; Kashii et al., 2019). Bisphosphonate treatment in our cohort improved the BMD of the OI patients, in particular in the 10–15 age-range. We noticed that although it was not specifically assessed in their work, the treatment responses (their Figure 1) in (Astrom and Soderhall, 2002) demonstrated highly similar patterns as ours, with sharpest responses in the 10–15 age-range. The effects of BP treatment on the height, however, was trivial. In fact, height growth rate at 15–20 appears to be lower than expected in the BP treated patients (Figure 4E). A study showed that children with OI receiving BP treatment have their height Z-scores decrease from -4.8 to -5.1, (Palomo et al., 2015). Another previous study reported that pamidronate therapy increased the Z-scores of heights in type III OI patients after 1 year treatment, but not in type I and type IV groups. After 4 years of treatment, only the Z-scores from type IV patients was improved (Zeitlin et al., 2003). In this regard, the application of bisphosphonate therapy in paediatric patients with low bone mass remains controversial (Bachrach and Ward, 2009).

In summary, our study detected known and novel mutations in *COL1A1* and *COL1A2* in OI patients of southern Chinese origin, revealed genotype-phenotype correlation and diversification, and assessed age-specific treatment response of bisphosphonate therapy. A novel splicing event was validated by functional assays. Histological and transcriptomic analyses offered some insights on bone microstructure and osteoblast differentiation. Further studies are needed to characterize the actual prevalence of OI in more representative cohorts, consummate the mutational spectrum both in dominant and recessive forms of OI, decipher the complicated connection between genotypes and phenotypes, and develop more effective therapeutic pharmacies and interventions.

## Abbreviations

BMD, bone mineral density; IQR, inter-quartile range; DEG, differentially expressed gene; OI, osteogenesis imperfecta; OI-COL1, OI patients carrying mutations on COL1A1 or COL1A2; OI-nonCOL1, OI patients not carrying mutations on COL1A1 or COL1A2; PCA, principal component analysis.

## Data Availability

The Sillence subtyping, genotypes, variant types and anonymized patient IDs were attached in Supplementary Table S1. The transcriptome data was deposited on NCBI GEO (accession: GSE186141). Scripts for processing the data and producing the figures are deposited on: https://github.com/HKUSZH/COL1.

## Supplementary Material

Supplementary Figure S1 DEGs between control and OI-COL1.

Supplementary Figure S2 Growth curves and inter-group comparisons.

Supplementary Figure S3 Growth curves and treatment responses.

Supplementary Figure S4 Radiographic images of eight patients carrying COL1A1 c.2461G>A, p.Gly821Ser mutation.

Supplementary Table S1 A full list of the 187 patients and their genetic information.

Supplementary Table S2 Clinical features of patients with splicing mutations selected for validation.

Supplementary Table S3 A full list of the 206 differentially expressed genes between the two non-OI samples and the six COL1 OI samples.
