# Supplementary material for: Phenotypic Spectrum and Molecular Basis in a Chinese Cohort of Osteogenesis Imperfecta With Mutations in Type I Collagen
Source: Front Genet. 2022 Jan 28;13:816078. doi: 10.3389/fgene.2022.816078 (PMC8831862; doi:10.3389/fgene.2022.816078)
Supplement: Supplementary file 3 [file DataSheet1.pdf]

Figure S1

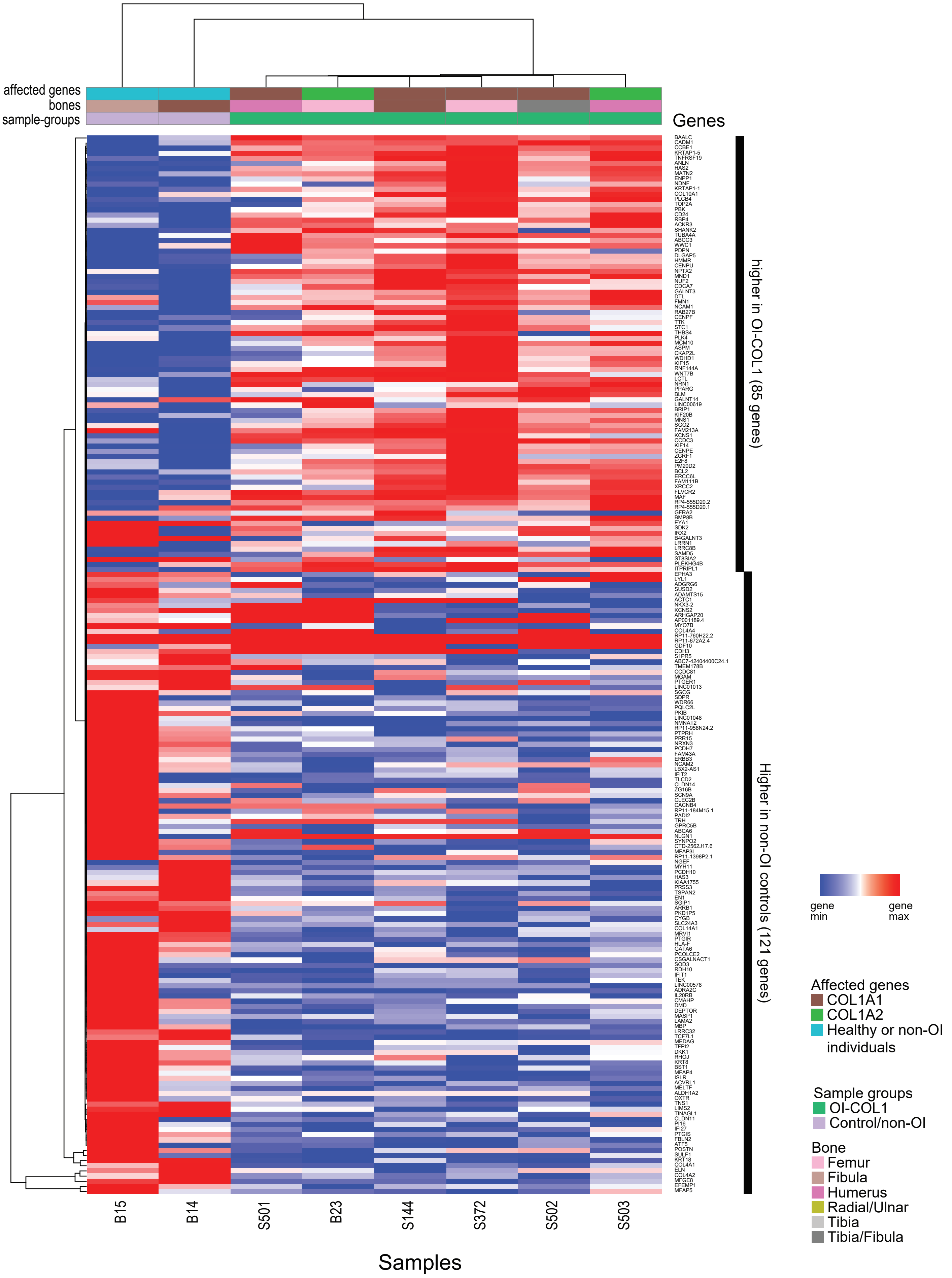

Figure S1. DEGs between control and OI-COL1.
Heatmap showing the gene-expression level (in logarithmic scale of FKPM) of DEGs between the 2 control (non-OI) samples and the 6 COL1A1/2 OI samples.

Figure S2

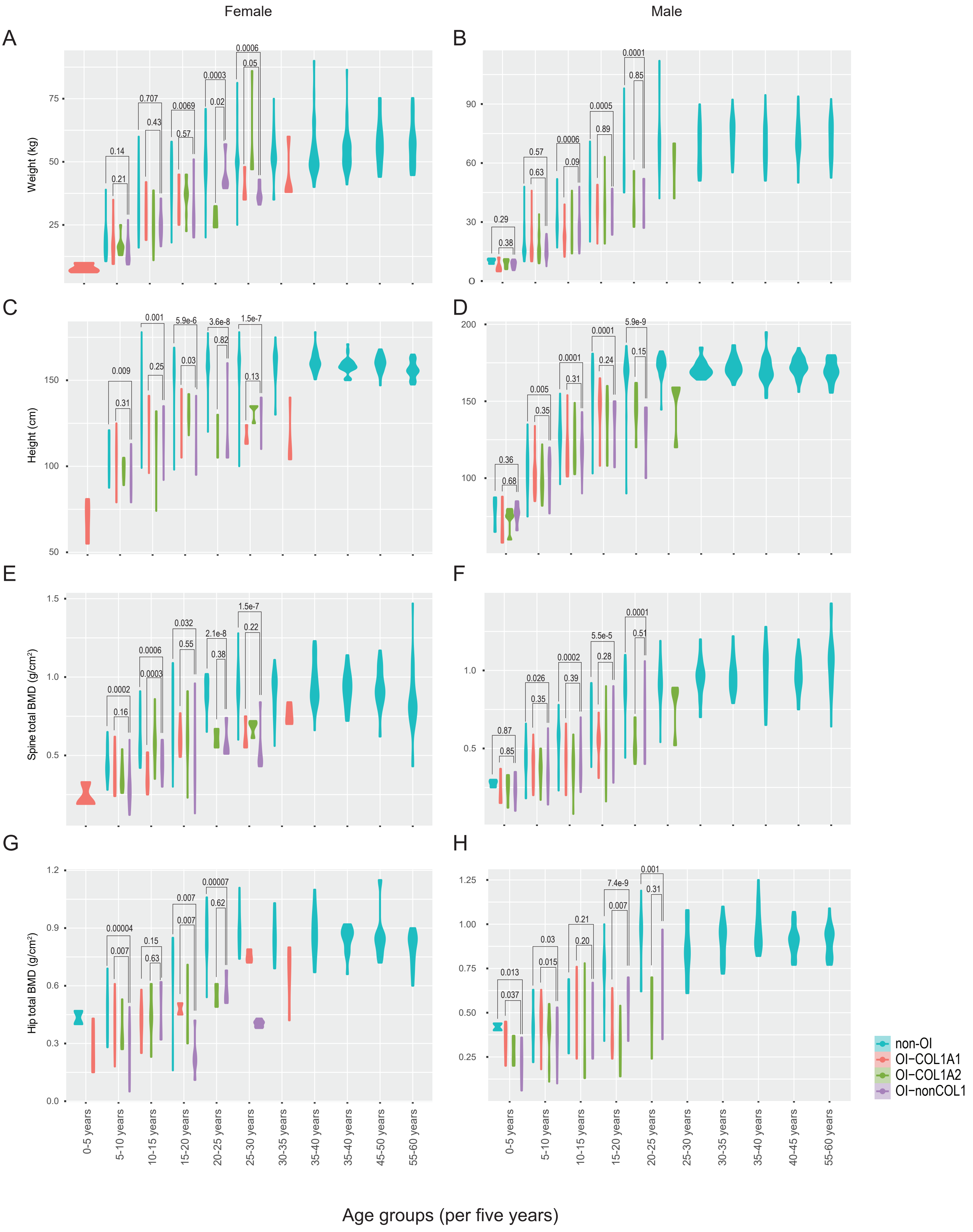

Figure S2. Growth curves and inter-group comparisons.

A, Violin plots showing the distribution for weight (**A-B**), height (**C-D**), spine BMD (**E-F**), and hip BMD (**G-H**), in a gender-specific manner (A, C, E, G for female, and B, D, F, H for male). The numbers on top of lines indicate χ^2 p-values for the analysis of variance (ANOVA) test among the four, three or two groups encompassed.

Figure S3

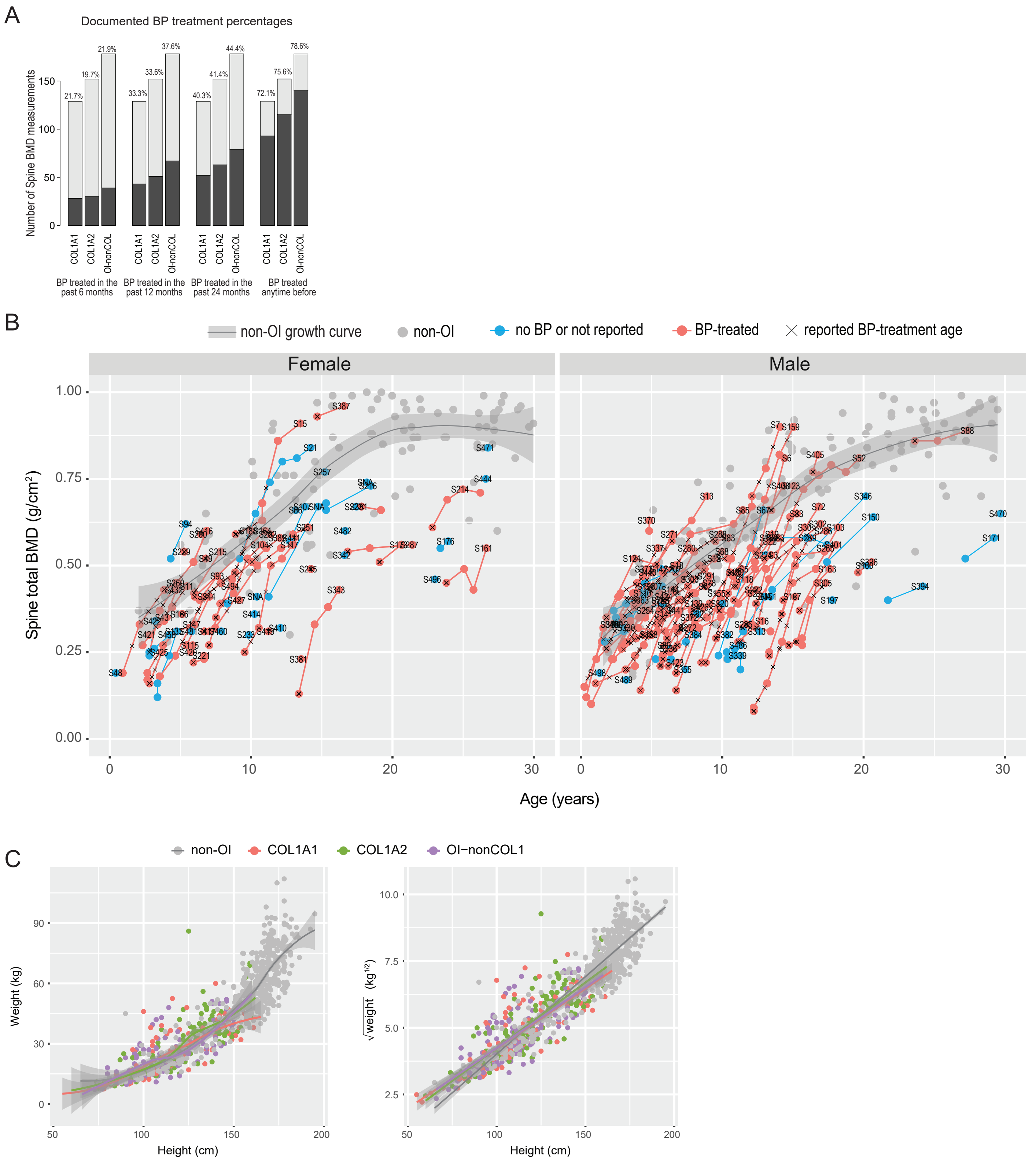

Figure S3. Growth curves and treatment responses.
A, Barchart showing percentage of patients with previous bisphosphonate treatment.
B, Scatter plots showing the BP treatment (in black crosses), in connection to the spine BMD data. The x-axis of the black crosses were derived from reported dates subtracting the birthdates, while the vertical axes were purely fitted based on linear relations of the BMD data. BP treatments before each person's first BMD measurements were represented with a single cross on the first BMD reading.
C, Scatter plots showing the relation between height and weight (left), and between height and square root of weight (right) in the cohort.

Figure S4

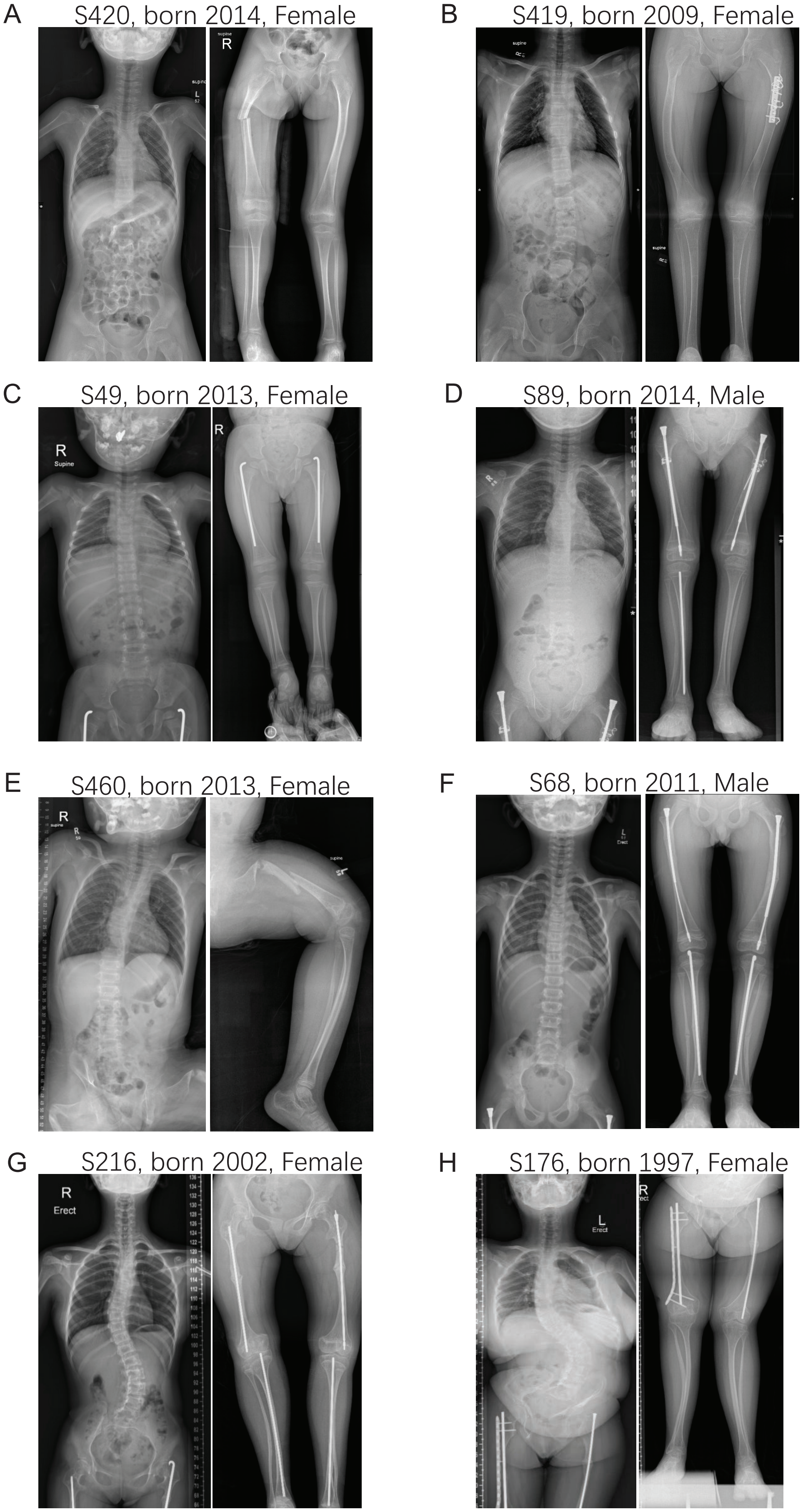

Figure S4. Radiographic images of 8 patients carrying COL1A1 c.G2461A, p.G821S mutation, as shown in Fig. 1C. X-ray images showing spine (left) and lower limbs (right) of each patient, with variable severity in clinical manifestation.
